# Experimental and Numerical Study of Membrane Residual Stress in Q690 High-Strength Steel Welded Box Section Compressed Member

**DOI:** 10.3390/ma17102296

**Published:** 2024-05-13

**Authors:** Jie Wang, Aimin Xu, Jin Di, Fengjiang Qin, Pengfei Men

**Affiliations:** 1 College of Civil Engineering and Architecture, Zhejiang University, Hangzhou 310058, China; wangjie205421@163.com; 2 High Grade Highway Construction Management Center of Ningbo City, Ningbo 315192, China; 13335746588@139.com; 3 Key Laboratory of New Technology for Construction of Cities in Mountain Area, School of Civil Engineering, Chongqing University, Chongqing 400045, China; qinfengjiang@cqu.edu.cn (F.Q.); menpengfei185811@163.com (P.M.)

**Keywords:** high-strength steel, welded box section, membrane residual stress, sectioning method, finite element model

## Abstract

High-strength steel (HSS) members with welded sections exhibit a notably lower residual compressive stress ratio compared with common mild steel (CMS) members. Despite this difference, current codes often generalize the findings from CMS members to HSS members, and the previous unified residual stress models are generally conservative. This study focuses on the membrane residual stress distribution in Q690 steel welded box sections. By leveraging experimental results, the influence of section sizes and welding parameters on membrane residual stress was delved into. A larger plate size correlates with a decrease in the residual compressive stress across the section, with a more pronounced reduction observed in adjacent plates. Additionally, augmenting the number of welding passes tends to diminish residual stresses across the section. Results showed that membrane residual stress adhered to the section’s self-equilibrium, while the self-equilibrium in the plates was not a uniform pattern. A reliable residual stress simulation method for Q690 steel welded box sections was established using a three-dimensional thermal–elastic–plastic finite element model (3DTEFEM) grounded in experimental data. This method served as the cornerstone for parameter analysis in this study and set the stage for subsequent research. As a result, an accurate unified residual stress model for Q690 steel welded box sections was derived.

## 1. Introduction

Owing to its lightweight and high strength, high-strength steel (HSS) is widely applicable in engineering structures. It can reduce component size, welding workload, and coating material consumption, as well as minimize the seismic response of structures and the required bearing capacity of foundations [1]. Longitudinal residual compressive stress notably impacts the buckling strength of axially compressed members in welded sections [2]. Previous studies [3,4,5] have demonstrated that the residual compressive stress ratio (ratio of residual compressive stress to the yield strength of the base metal) in HSS welded members is significantly lower than that in common mild steel (CMS) welded members. However, current specifications [6,7,8] often apply the residual compressive stress values of CMS welding sections to HSS welding sections, leading to the underutilization of the strength of HSS. Q690 steel is the highest strength steel allowed to be used in the current steel structure design specifications [6,7,8]. The membrane residual stress discussed in this study is macroscopic residual stress.

The experimental measurement methods for residual stress encompass various techniques, including the blind hole method, sectioning method, indentation method, ultrasonic method, magnetic measurement method, X-ray diffraction method, neutron diffraction method, and synchrotron radiation diffraction method [9]. Among these methods, the blind hole method, indentation method [10], and X-ray diffraction method [11] excel at obtaining accurate measurement results for the residual stress distribution on the surface of members. Neutron diffraction and synchrotron radiation diffraction methods can measure residual stress inside components without causing damage, but they require large scientific devices and are limited by the size of the member being tested [9]. The accuracy of ultrasonic testing is relatively lower [12], and the magnetic method [13] is unable to test residual stresses above 300 MPa. Considering factors such as feasibility, accuracy, and cost, the sectioning method emerges as a widely employed technique for measuring membrane residual stresses in engineering structures.

Rasmussen and Hancock [14,15] conducted a sectioning method to measure the membrane residual stress in a 690 MPa steel welded box section, aiming to identify initial imperfections in the compressed members. Ban et al. [4,16] performed a sectioning method to study the membrane residual stress in Q460 and Q960 steel welded box sections. The findings showed that the plate’s membrane residual compressive stress is related to the section size, but not to the steel strength. By integrating existing test results, a unified model for membrane residual stress in HSS welded box sections was proposed. Somodi et al. [5] measured the membrane residual stress in S235 to S960 steel welded box sections using the sectioning method and proposed a unified membrane residual stress model based on the test results. Yang et al. [17] employed the sectioning method to measure the residual stress in thick-walled box sections welded with Q460GJ steel. Subsequently, they developed a simplified multilayer residual stress model. Khan [18] investigated the residual stress in both heavy and light welded box sections with S690 steel using the neutron diffraction method and proposed a simplified residual stress model applicable to both. Clarin et al. [19], Wang et al. [20], Li et al. [3], and Cao et al. [21] measured the membrane residual stress in 460, 690, 800, and 1100 MPa HSS welded box sections and proposed simplified residual stress models accordingly to account for initial imperfections in the compressed members.

Conducting experimental studies on the membrane residual stress in welded sections is cost intensive, and experimental measurement methods still have inherent limitations regarding spatial resolution, measurement accuracy, and testing conditions [22]. In the early 1970s, a “thermal–elastic–plastic” finite element method was developed for predicting welding residual stress [23,24]. This analytical approach combines thermal conduction finite element calculation with structural elastic–plastic finite element calculation. With the rapid advancement of computer technology, the “thermal–elastic–plastic” finite element method is increasingly applied to calculate welding deformation and residual stress in large and complex engineering structures. Wang [25] utilized a “three-dimensional thermal–elastic–plastic finite element model” (3DTEFEM) to simulate residual stress in Q460 steel welded box sections and validated the model against the test results. However, this 3DTEFEM was not employed for parameter analysis. Zhang et al. [26] employed the “thermal–elastic–plastic” finite element method to investigate residual stress in butt-welded joints involved in complex column–beam welded structures with Q390 steel. Subsequently, based on the “thermal–elastic–plastic” finite element method, the metal phase transformation process was considered, resulting in the development of a “thermal–metallurgical–mechanical” finite element method [27,28,29,30]. This method is primarily applied to the study of macroscopic residual stress [31,32,33,34].

Current research on membrane residual stress in a Q690 steel welded box section compressed member lacks dependable finite element models for extensive parameter analysis, and existing unified residual stress models are generally conservative. This study bridged this gap by establishing a reliable 3DTEFEM grounded in experimental data. By leveraging experimental and finite element parameter analysis results, this study conducted in-depth research on the influence mechanism of section sizes and welding parameters, and an accurate unified residual stress model was derived.

## 2. Experimental Process

### 2.1. Specimens

**Figure 1:**
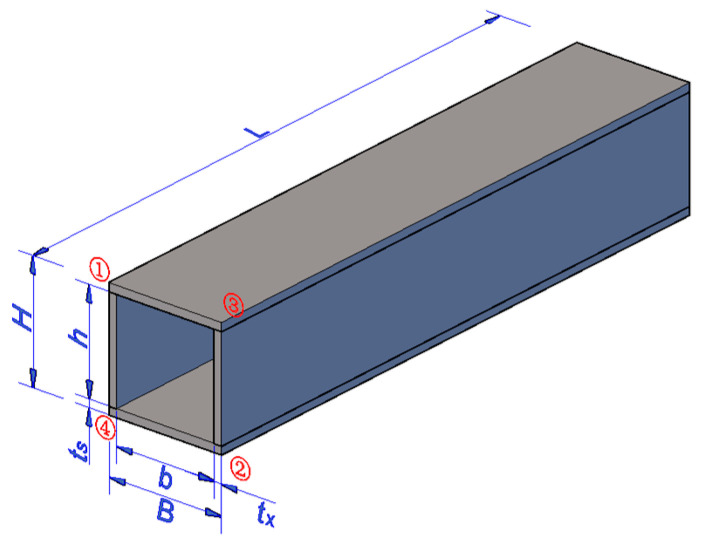
Schematic representation of the specimen.

Five specimens were designed to study the membrane residual stress in Q690 steel welded box sections. The construction and parameters of the specimen is illustrated in Figure 1. The number “①” in figures represents welding sequence. The mean values (MV) and standard deviations (SD) of the measure dimensions of these specimens are shown in Table 1. In the specimen name, “RB” denotes a membrane residual stress specimen with a box section, while the following number, like “1”, is used to distinguish section of the specimen. Specimen RB-1 served as the reference specimen. The nominal width and thickness of the RB-1 plate are 204 mm and 12 mm, respectively. One specimen was designed for each parameter to examine the impacts of the flange width, the web width, and the flange thickness. RB-5 was designed for examining the impacts of the number of welding passes, because the number of welding passes is an important parameter in the welding process, which directly affects the heat input. To mitigate the end effect, the minimum length of each specimen was set at 290 mm plus four times of the maximum of the cross-sectional width and height [35,36].

Plasma cutting, which has a smaller thermal deformation and heat-affected zone than flame cutting, was used to cut the steel plate. Carbon dioxide shield welding was employed to weld the plates. Full penetration welding with a unilateral 45-degree groove on the web was used for all specimens. An optimized welding sequence, as indicated by the numbers in Figure 1, was adopted to minimize welding deformation. The average welding voltage and current were approximately 31.5 V and 240 A, respectively. The welding speed is the quotient of the length of the weld seam and the welding time, and welding time does not include intermediate pause time. The two welding speeds for two-pass welding were 5.4 mm/s and 4.6 mm/s, respectively, while the welding speed for single-pass welding was 3.1 mm/s. The welding wire used for Q690 steel was CHW-80C, fabricated by Atlantic China Welding Consumables, Inc. (Zigong, China), complying with the AWS A5.28M-2005 [37] and GB/T 8110-2020 [38], being ER110S-G and ER76-G, respectively. The chemical composition and mechanical properties of the welding wire are illustrated in Table 2 and Table 3, respectively. The mechanical properties of deposited metal come from the qualification certificate of welding wire. The preparation and testing of deposited metal specimens are in accordance with GB/T 25774.1-2023 [39] and GB/T 228.1-2021 [40].

**Figure 2:**
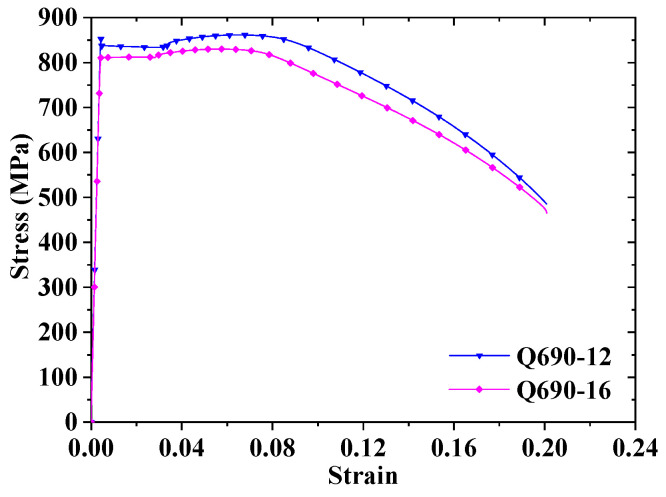
Stress–strain curves of various steel plates.

The test utilized two Q690 steel plates with thicknesses of 12 and 16 mm fabricated by Wuhan Iron and Steel Co., Ltd. (Wuhan, China); their chemical composition is illustrated in Table 4. Tensile coupons were manufactured and tested in accordance with GB/T 2975-2018 [41] and GB/T 228.1-2021 [40]. The MV and SD of mechanical properties for each steel plate were determined based on the test results from three tensile coupons, as depicted in Table 5. The average stress–strain curves are illustrated Figure 2. In the steel plate designation “Q690-12”, “Q690” indicates the steel grade and “12” refers to the plate’s thickness. *ε*_st_ represents the strain at the end of the yield plateau, while *ε*_u_ represents the strain at the tensile stress *f*_u_. The stress–strain curves of both steel plates show clear yield platforms. The yield strength, tensile strength, and elongation after fracture meet the requirements of specification [6], and their lower limits are 690 MPa, 770 MPa, and 16%, respectively. However, the yield ratio *f*_y_/*f*_u_ does not; the requirement of specification [6] is less than 0.9.

### 2.2. Sectioning Process

**Figure 3:**
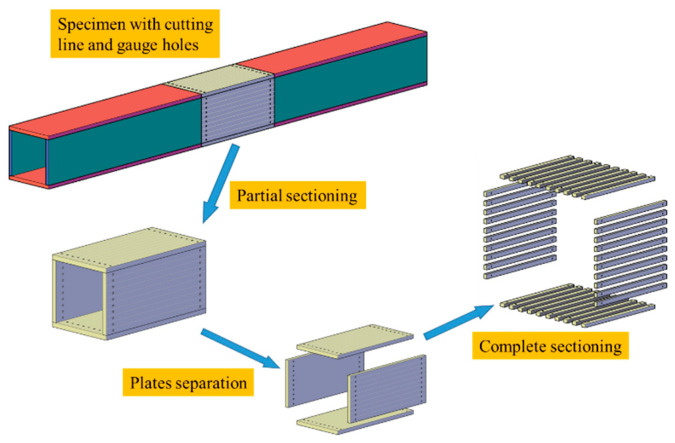
Sectioning process of specimen.

**Figure 4:**
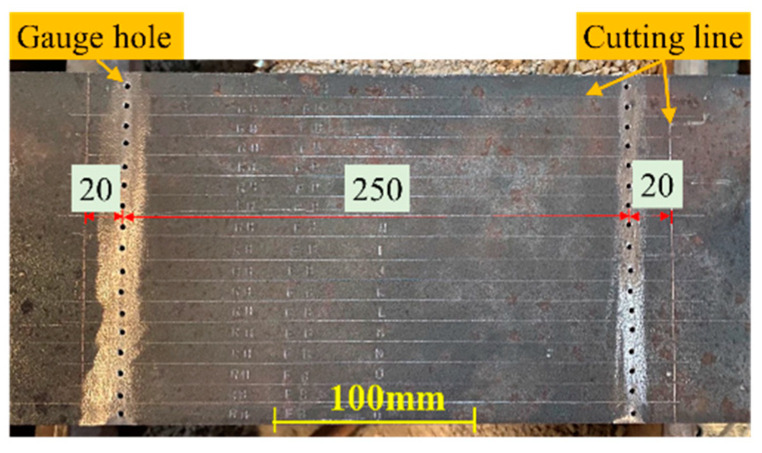
Specimen with cutting line and gauge holes (unit: mm).

**Figure 5:**
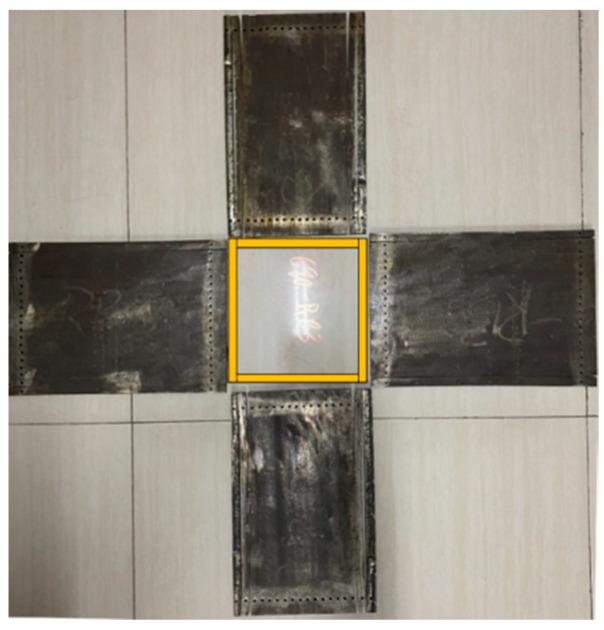
RB-1 strips after complete sectioning.

The residual stress in the specimens was measured using the sectioning method. Figure 3 illustrates the sectioning process. The steps for measuring residual stress are outlined as follows:

1. Measurement before partial sectioning: It was essential to mark the cutting lines and drill gauge holes before measuring. Figure 4 displays a specimen marked with cutting lines and gauge holes. The initial gauge length was measured using a Whittemore strain gauge. Three sets of gauge length measurements were recorded, with discrepancies between them being less than 0.01 mm. Measurements were taken on both surfaces of the plates. To eliminate deviations due to temperature variation, the gauge length of a reference strip was measured. Then, the sectioning zone, as marked in step 1, was cut from the specimen using a sawing machine.
2. Measurement before plate separation: Since the hole spacing inside the box could not be measured prior to partial sectioning, an additional measurement step, identical to the method described in step 2, was conducted. Then, the sectioning zone was divided into individual plates using a wire-cut electron discharge machine (WEDM).
3. Measurement before complete sectioning: The gauge lengths of each plate’s strips were measured as in step 2 for two main reasons: Firstly, it was impossible to measure the inner-side distance of the strips at the plate junctions prior to separating the plates. Secondly, the results from this step could be refined using the self-equilibrium of the plate. Then, strips were cut from the plates using a WEDM, with most strips being 10 mm wide. However, strips from the compressive zone of the wide plate were 20 mm wide. Figure 5 exemplifies this by displaying RB-1’s strips post-sectioning.
4. Measurement after complete sectioning: Gauge lengths for the strips from each plate were measured, following the same procedure as in step 2. Additionally, bending deformations in curved strips were measured after sectioning.

The Whittemore strain gauge had a precision of 0.001 mm. The vernier caliper offered a precision of 0.01 mm. Additionally, the dial gauge employed for bending deformation measurement provided a precision of 0.001 mm.

## 3. Test Results

### 3.1. Measured Residual Stress

Distances between gauge holes in each strip for steps two, four, six, and eight are labeled as *l*_1_, *l*_2_, *l*_3_, and *l*_4_. Corresponding distances in the reference strip are noted as *l_t_*_1_, *l_t_*_2_, *l_t_*_3_, and *l_t_*_4_. Residual stress from partial sectioning is denoted as Δ*σ*_1_. During plate separation, the resulting residual stress is denoted as Δ*σ*_2_. The stress Δ*σ*_2_ on the inner side of the strips at junctions is inferred from the residual stress on the outer side, considering the residual stress relationships and self-equilibrium of the section. Residual stress from complete sectioning is labeled as Δ*σ*_3_. Post-sectioning, the strip near the weld seam exhibits notable in-plane or out-of-plane bending, with the arc bending stress, Δ*σ*_c_, determined using Sherman’s Equation (4) [42]. The final residual stress, denoted as *σ*_rs_, is calculated with Equations (1)–(3) [1,3,17,21]:

(1) $$\left\{\begin{array}{l} \Delta \sigma_{1}=\frac{\left(l_{1}-l_{t1})-(l_{2}-l_{t2}\right)}{250}\times E\\ \Delta \sigma_{2}=\frac{\left(l_{2}-l_{t2})-(l_{3}-l_{t3}\right)}{250}\times E\\ \Delta \sigma_{3}=\frac{\left(l_{3}-l_{t3})-(l_{4}-l_{t4}\right)}{250}\times E \end{array}\right.$$

(2) $$\Delta \sigma_{c}=\frac{8}{3}E\times {\left(\frac{\delta }{250}\right)}^{2}$$

(3) $$\sigma_{rs}=\Delta \sigma_{1}+\Delta \sigma_{2}+\Delta \sigma_{3}-\Delta \sigma_{c}$$

where *δ* denotes the arch rise of the curved strips, and *E* is the measured elastic modulus (see Table 5).

**Figure 6:**
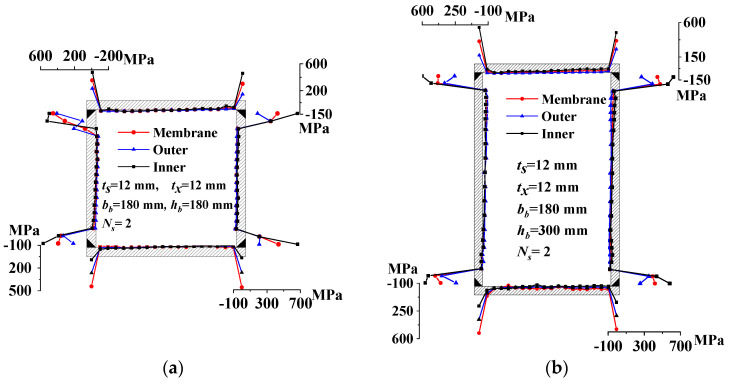

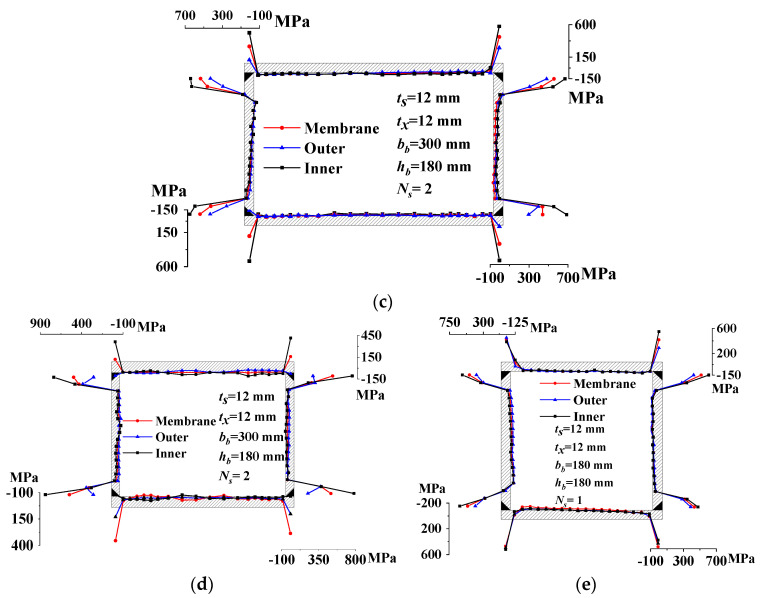
Measured residual stress: (**a**) RB-1; (**b**) RB-3; (**c**) RB-2; (**d**) RB-4; and (**e**) RB-5.

Figure 6 displays the welding residual stress in all specimens. Membrane stress refers to the average longitudinal residual stress across the plate’s thickness. The web’s residual tensile stress, both in value and distribution range, exceeds that of the flange, attributed to its ends being subject to full penetration welding. It is consistent with the test results in Ref. [16]. The web’s residual tensile stress spans 22 mm at both ends, whereas the flange’s residual tensile stress zone extends 12 mm at both ends. Residual compressive stress occupies the remaining areas. Notably, there’s a marked difference in residual tensile stress between the inner and outer sides, while the residual compressive stress is comparatively uniform across these areas. The welding sequence induces a slight gradient in the residual compressive stress distribution across the plate’s width, with higher stress observed around the front weld seam. Ref. [20] obtained the same test result.

### 3.2. Self-Equilibrium of Section

The unbalanced force (*F*_err_) and unbalanced stress (*σ*_err_) can be calculated using the following equation [16,21]:

(4) $$\left\{\begin{array}{l} F_{err}=\sum_{i=1}^{n}(\sigma_{rsi}\times A_{i})\\ \sigma_{err}=F_{err}/A \end{array}\right.$$

where *σ*_rsi_ denotes the membrane residual stress of each strip, and *A_i_* is the cross-sectional area of each strip, and *n* represents the number of strips.

Table 6 presents the unbalanced forces for both the plates and the specimens. Except for specimen RB-3, the plates’ unbalanced forces were notably above zero, suggesting a lack of uniform self-equilibrium of the plates and an interaction between plates. In contrast, the unbalanced forces across the entire sections of all specimens were near zero, demonstrating their compliance with the self-equilibrium criteria for the entire section, as shown in the test results of Refs. [5,20]. The maximum unbalanced stress across the entire section reached only 7.24 MPa.

The welding heat input in the web exceeded that of the flanges, as the full penetration welding zone was located at the web’s ends. The web experienced significant welding deformation, while the flange primarily served to restrain this deformation. Change in plate size led to corresponding alterations in welding deformation, which in turn affected the constraint force (or unbalanced force) between the plates. Consequently, the web’s unbalanced force manifests as tensile force, whereas the flange exhibit pressure force. The absolute value of the plate’s unbalanced force diminishes with widening web width and intensifies with increasing width and thickness of the flange. An increase in welding passes amplifies welding deformation and the constraint force between plates, thereby elevating the absolute value of the plate’s unbalanced force.

### 3.3. Simplified Model of Membrane Residual Stress

To enable comparative analysis and application, the measured membrane residual stress is simplified [1,3,5,17,21]. The primary use of the measured membrane residual stress is for the compression member. In the simplified model, the value and distribution of membrane residual compressive stress are represented with maximum accuracy. Calculation of the membrane residual tensile stress in the welding zone is based on the self-equilibrium of the entire section.

**Figure 7:**
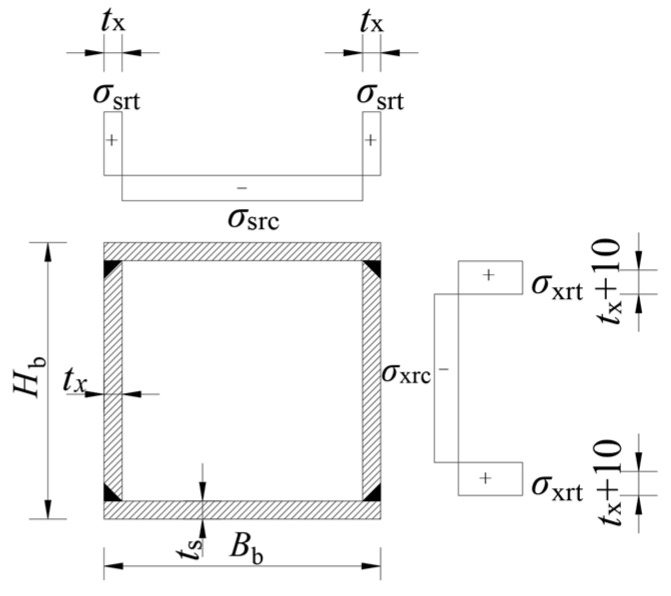
Simplified membrane residual stress model (unit: mm).

Based on the membrane residual stress measurements in Figure 6, a 22 mm zone at both ends of the web and a 12 mm zone at both ends of the flange are identified as their respective membrane residual tensile stress zones. The plates’ remaining areas are designated as the residual compressive stress zones, lacking any transition sections [3,20]. The mean values and standard deviations of the measured membrane compressive residual stress values of all strips in the plates are derived. Membrane residual tensile stress is calculated based on the self-equilibrium of the entire section, ensuring the peak stress ratio remained constant. The mean values and standard deviations of the measured membrane tensile residual stress values are derived. Figure 7 and Table 7 illustrate the simplified models of membrane residual stress, where *σ*_srt_ and *σ*_src_ denote the flange’s tensile and compressive residual stresses, respectively, and *σ*_xrt_ and *σ*_xrc_ represent the same for the web.

Table 7 indicates that a 67% increase in the width of the flange leads to an approximate 9% and 13% decrease in *σ*_src_ and *σ*_xrc_, respectively. As the web’s width increases by 67%, *σ*_src_ decreases by approximately 46%, while *σ*_xrc_ remains largely unchanged. An increase in the flange’s thickness from 12 mm to 16 mm results in *σ*_src_ and *σ*_xrc_ decreasing by approximately 35% and 24%, respectively. A reduction in welding passes from 2 to 1 leads to an approximately 26% and 47% increase in *σ*_src_ and *σ*_xrc_, respectively.

The membrane residual tensile stress in the simplified model closely matched the measured values. A 67% increase in the flange width results in an approximately 9% and 28% increase in *σ*_srt_ and *σ*_xrt_, respectively. With a 67% increase in the web width, *σ*_srt_ rises by approximately 17%, while *σ*_xrt_ remains largely stable. An increase in flange thickness from 12 mm to 16 mm leads to a roughly 63% decrease in *σ*_srt_, with *σ*_xrt_ remaining relatively constant. A decrease in welding passes from 2 to 1 corresponds to an approximately 51% and 15% increase in *σ*_srt_ and *σ*_xrt_, respectively.

**Figure 8:**
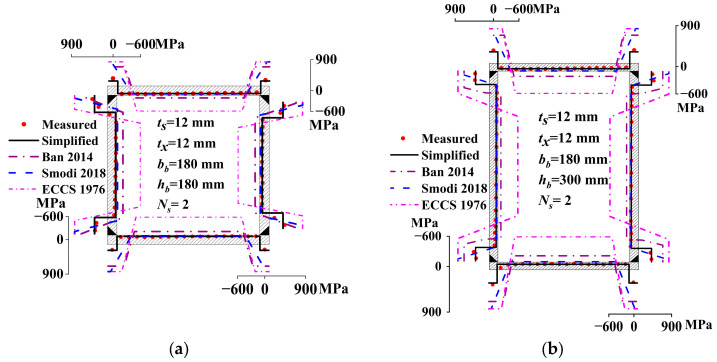

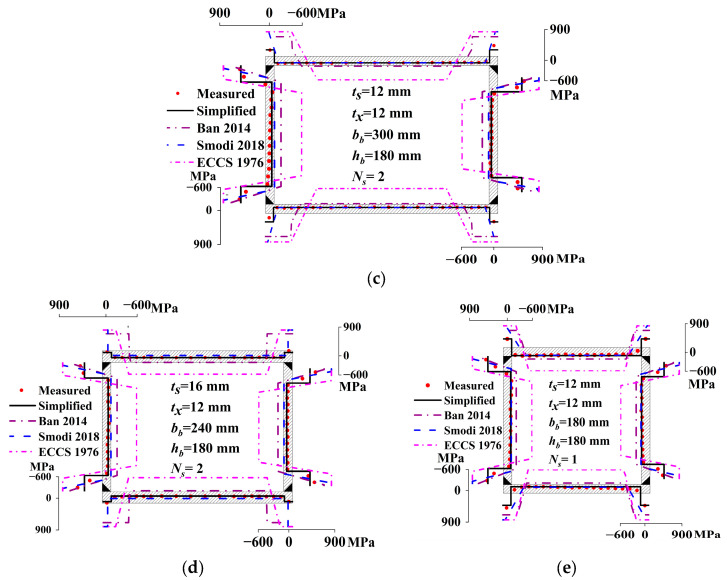
Comparison of the measured value with simplified and existing models [1,5,43]: (**a**) RB-1; (**b**) RB-3; (**c**) RB-2; (**d**) RB-4; and (**e**) RB-5.

Figure 8 compares the measured residual stress with the simplified residual stress models from this study and references [1,5,43] to evaluate their applicability to Q690 steel. The simplified model in this study shows good agreement with the measured residual stress. The tensile and compressive residual stress of the simplified models in Refs. [1,43] significantly exceeds the measured value, making it overly conservative. The residual compressive stress in Refs. [1,43] is, on average, 3.4 and 9.6 times of the measured value, respectively. The simplified model in Ref. [5] exhibits relatively high residual tensile stress, but its residual compressive stress aligns well with the measured value; it is, on average, 1.5 times the measured value. However, this model is applicable only to plates with thicknesses under 12 mm.

## 4. Unified Residual Stress Model Based on FEM

### 4.1. Finite Element Model

A 3DTEFEM was established using ANSYS software version 14.5 to simulate the residual stress in the Q690 steel welded box section. The FEA comprised temperature and stress field analysis. FEA and prior studies [35,36] indicate that when a member’s length exceeds three times its maximum cross-sectional dimension, its midsection’s welding residual stress remains unaffected by end effect. Consequently, the length of 3DTEFEM was set at 3 × Max(*H*, *B*) + 20 mm, with *H* and *B* representing the member’s cross-sectional height and width, respectively.

**Figure 9:**
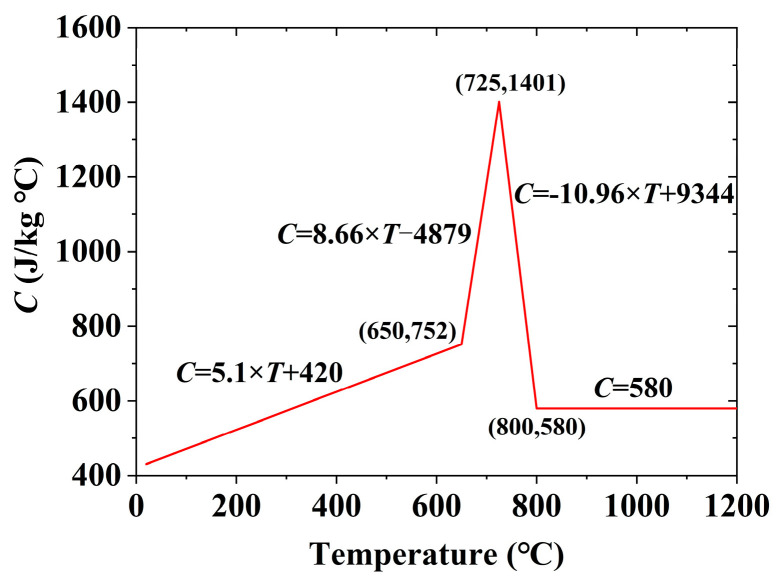
Specific heat in the 3DTEFEM of welding residual stress.

**Figure 10:**
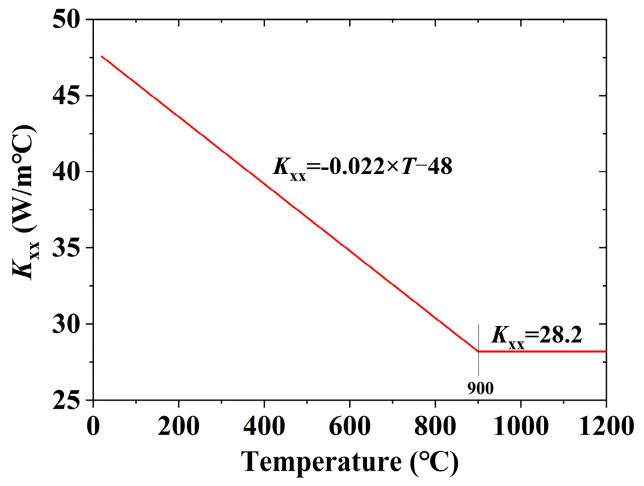
Thermal conductivity in the 3DTEFEM of welding residual stress.

For temperature field analysis, the 3D thermal solid element Solid70, featuring eight nodes with a single temperature degree of freedom each, was employed. This element is suitable for both steady-state and transient 3D thermal analysis. The grid sizes for the weld seam’s cross-section and other areas were set to 1 mm and 5 mm, respectively. In the longitudinal direction, the grid size equated to the welding length per second. During the temperature field analysis, the specific heat (*C*) and thermal conductivity (*K*_xx_) values for steel at various temperatures followed the recommendations in ASCE’s No. 78 engineering practice manual “Structural Fire Protection,” as illustrated in Figure 9 and Figure 10 [44]. The boundary condition for the temperature field analysis involved thermal convection with the surrounding environment. The reference temperature was set at the ambient welding temperature of approximately 25 °C. The heat exchange coefficient was notably high at 2.5 × 10^−5^ W/(mm^2^·s), because a fan was used to accelerate airflow. The welding process simulation employed the birth and death element method to ascertain the heat input. Heat input calculation employed the Equation (5). Carbon dioxide gas shielded welding’s thermal efficiency ranges from 0.65 to 0.85 [45]. The specific value was ascertained based on the 3DTEFEM verification results.

(5) K=ηUI

where *η* denotes the welding thermal efficiency, and *U* and *I* represent the actual voltage and current of the welding arc, respectively.

**Figure 11:**
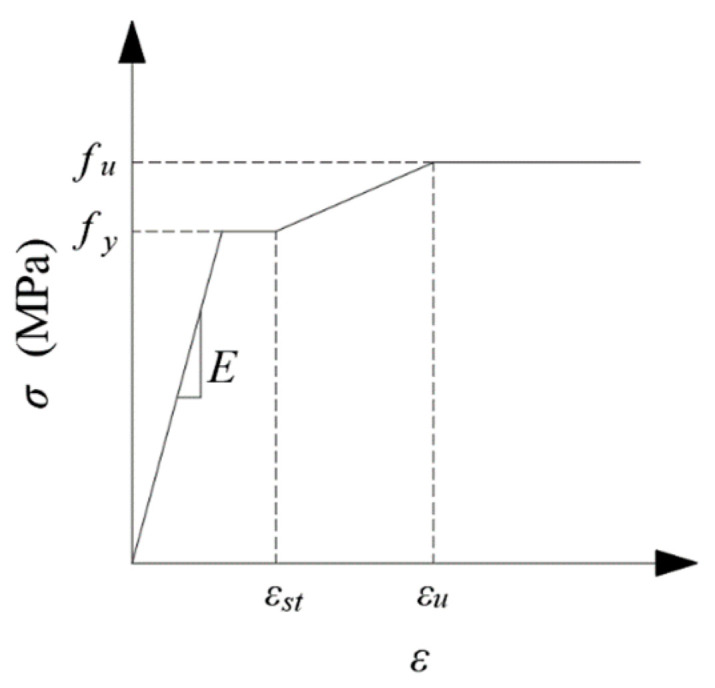
Stress–strain relationship: trilinear kinematic hardening model.

**Figure 12:**
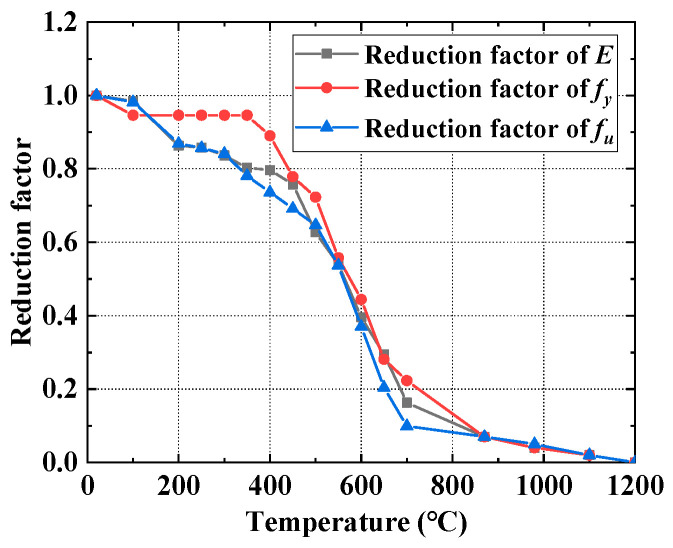
The reduction factors for elastic modulus, yield strength, and tensile strength.

**Figure 13:**
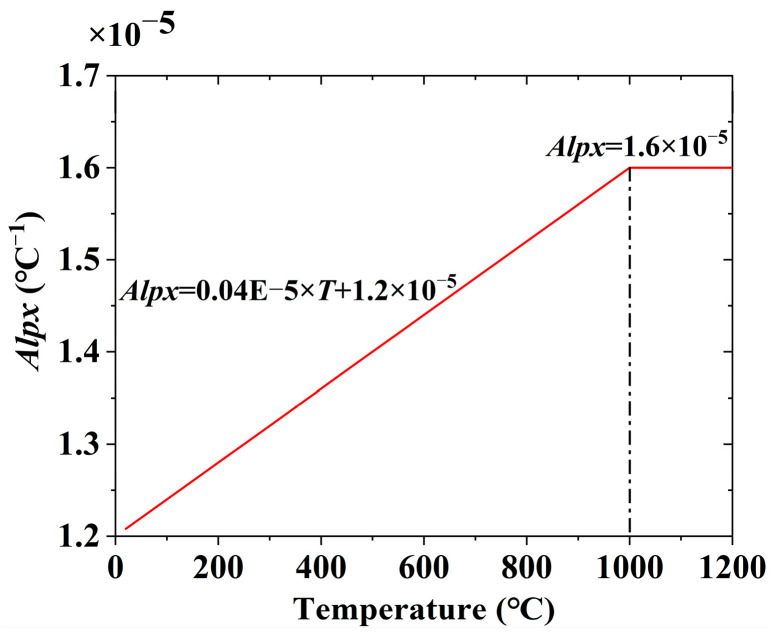
Coefficient of thermal expansion in the 3DTEFEM of welding residual stress.

**Figure 14:**
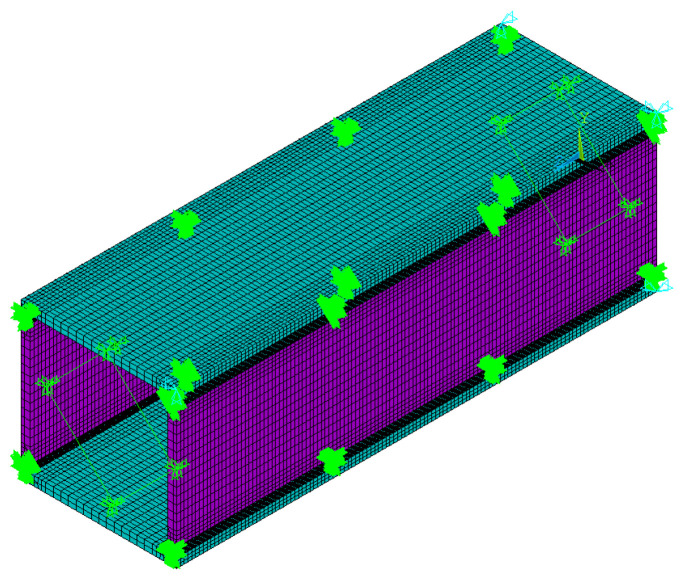
3DTEFEM for stress field analysis.

**Figure 15:**
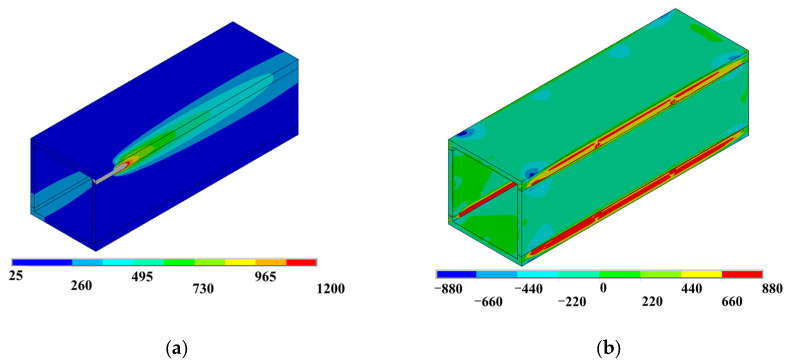
Simulation process of residual stress: (**a**) temperature field analysis (unit: °C); (**b**) stress field analysis (unit: MPa).

Stress field analysis was conducted based on the previously obtained temperature field data. For stress field analysis, the 3D eight-node structural element Solid185, derived from the Solid70 thermal element, was utilized. Each node of Solid185 possesses three translational degrees of freedom, exhibiting hyper elasticity, stress hardening, creep behavior, as well as large deformation and strain capabilities. The melting point of the steel was 1200 °C. For Q690 steel, a multilinear kinematic hardening model with a yield plateau, based on the von Mises yield criterion, was applied (refer to Figure 11). The reduction factors for the elastic modulus, yield strength, and tensile strength of Q690 steel below 700 °C were established based on the test results in Ref. [46]. This finding indicates that the reduction factors at elevated temperatures closely align with ANSI/AISC 360-22 [8], particularly when temperatures exceed 500 °C. Consequently, for temperature ranging from 870 to 1200 °C, the reduction factors for Q690 steel were defined in accordance with ANSI/AISC 360-22 guidelines. Meanwhile, for temperature between 700 and 870 °C, the reduction factors were determined through linear interpolation. The reduction factors for the elastic modulus, yield strength, and tensile strength of Q690 steel are depicted in Figure 12. Steel’s thermal expansion coefficient (*Alpx*) was sourced from ASCE’s No. 78 engineering practice manual “Structural Fire Protection”, as illustrated in Figure 13. Rigid connections simulated the spot welding of plates during pre-welding assembly and the diagonal brace setup to mitigate excessive welding deformation. The 3DTEFEM’s boundary condition was designed to restrict rigid displacement while allowing free deformation during welding. The birth and death element method was similarly employed to progressively simulate welding and establish the weld connection between plates. Figure 14 depicts the 3DTEFEM used in the stress field analysis. The temperature and stress field simulation processes are depicted in Figure 15.

### 4.2. Finite Element Model Verification

**Figure 16:**
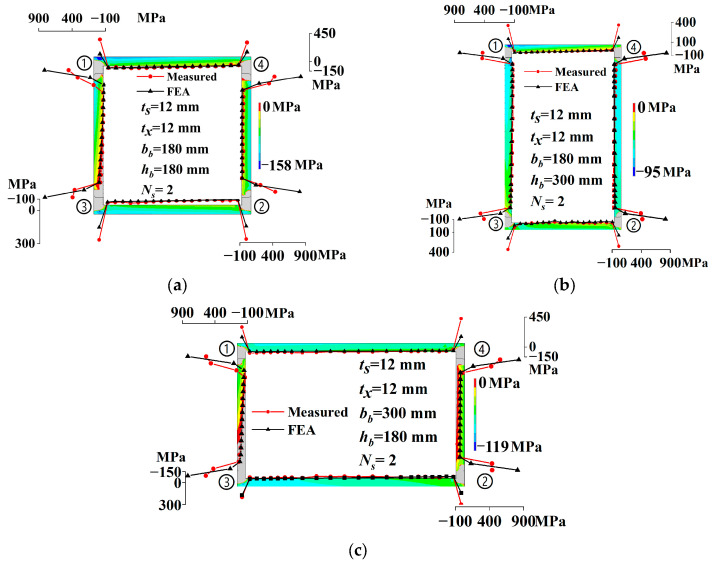

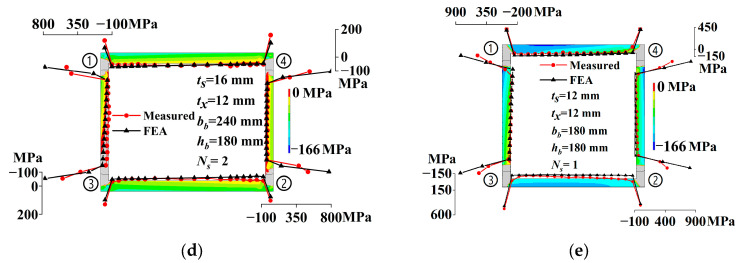
Comparison of the simulated and measured membrane residual stress of specimens: (**a**) RB-1; (**b**) RB-3; (**c**) RB-2; (**d**) RB-4; and (**e**) RB-5.

The proposed 3DTEFEM was employed to simulate the residual stresses of specimens. Simulated membrane residual stress was compared with measured value to validate the accuracy of 3DTEFEM. The extraction points for membrane residual stress in the finite element simulation matched the transverse position of the sectioning strip. Figure 16 presents a comparison between the simulated and measured membrane residual stresses. The distribution cloud maps of residual compressive stress of specimens were also depicted in Figure 16. The number “①” in figures represents welding sequence. To reflect the distribution gradient of residual compressive stress, the residual tensile stress is not given for its large value. Table 8 details the specific values of both measured and simulated membrane residual compressive stresses.

From the residual compressive stress distribution cloud maps of the cross-section in Figure 16, it can be seen that the residual compressive stress of the plate was not uniformly distributed in the thickness direction. Its value on the inner side was smaller than that on the outer side, especially when the plate width was small. When the plate size was the same, the distribution gradient of residual compressive stress in the thickness direction of the web was greater than that of the flange. In the residual compressive stress zone, residual tensile stress even appeared on the inner side. The residual compressive stress distribution area of the web was smaller than that of the flange. In the residual tensile stress zone of the flange, residual compressive stress still appeared on the outer side. Additionally, the residual compressive stress around the rear weld seam was smaller than that around the front weld seam.

Given the low membrane residual stress values in some specimens, it is more appropriate to assess agreement based on the difference in values, rather than the rate of difference. As illustrated in Figure 16 and Table 8, the discrepancy between measured and simulated membrane residual compressive stresses remained under 10% or within a 10 MPa range. The consistency between the measured and simulated membrane residual compressive stresses was notable. However, there was a significant deviation between the simulated and measured values of membrane residual tensile stress. This discrepancy could be attributed to the challenges associated with measurements in this specific position. With a calculated welding thermal efficiency of 0.72, the 3DTEFEM reliably simulates the membrane residual stress in Q690 steel welded box sections. This is particularly true for membrane residual compressive stress, which substantially affects member buckling.

### 4.3. Unified Residual Stress Model

As indicated in Section 3.3, the width and thickness of the flange and web significantly influence residual stress. Analyzing all these parameters demands substantial computer runtime. Consequently, this study employed the previously verified 3DTEFEM for parameter analysis of Q690 steel welded box sections within specific size constraints, demonstrating the practicality of the model. In this parameter analysis, the box section was defined with specific limitations: plate thickness was set at 12 mm, and the cross-section’s height and width were equal. The welding parameters were consistent with those used for the specimen in Section 2.1. For the parameter analysis, the width-to-thickness ratio of plate was established in the range of 15 to 60. The section sizes used in the parametric analysis are typical for engineering structures. The strength and ductility values of the Q690 steel in the simulation adhere to the limits specified in specification [6].

**Figure 17:**
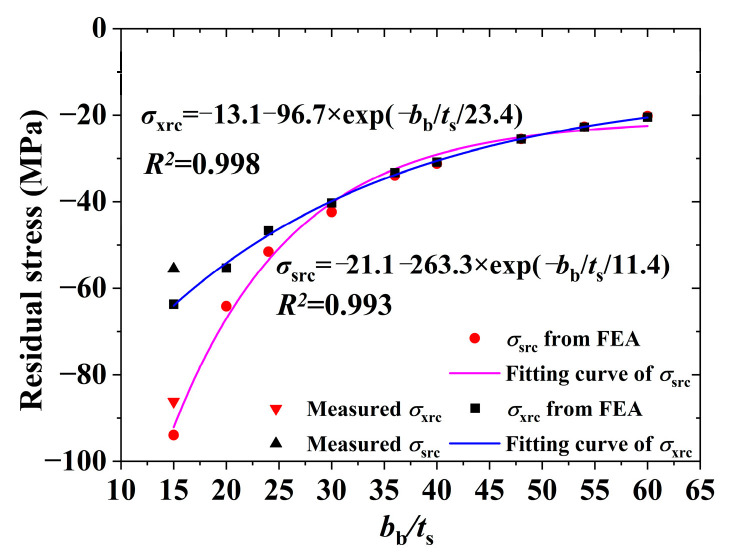
Residual compression stress from parametric analysis.

The welding residual stress from FEA was simplified using the method described in Section 3.3, with the distribution mode of this simplified residual stress model depicted in Figure 7. Figure 17 illustrates how the residual compressive stress (*σ*_src_ and *σ*_xrc_) of plates varies with their width-to-thickness ratio. The calculation formulas for these stresses were derived by fitting the data to a first-order attenuation exponential distribution. The *R^2^* value in the figure represents the goodness of fit. Based on the test results, the web’s residual tensile stress was uniformly set at 420 MPa to simplify application. The distribution zone for the residual tensile stress of web was confined to a 22 mm range at both ends. The residual tensile stress of the flange was determined based on the self-equilibrium of the entire section. It is concluded that the simplified residual stress model for Q690 steel welded box sections is applicable when plate thickness is 12 mm and the cross-sectional height and width are identical, as shown in Figure 7 and Table 9. In this study and previous research, only specimen RB-1 satisfies the section requirement of the proposed unified residual stress model, and its measured value of *σ*_src_ and *σ*_xrc_ are also depicted in Figure 17. It is evident from this data that the proposed unified residual stress model is applicable.

The proposed unified residual stress model plays a crucial role in calculating the bearing capacity of compressed members in engineering structures. It is advantageous for engineering structural designers to fully utilize the strength of Q690 steel while ensuring structural safety. Furthermore, it can promote the development of steel structure design specifications.

## 5. Conclusions

This study investigated the membrane residual stress of a Q690 steel welded box section compressed member using the sectioning method and FEA. The following conclusions were drawn:

1. The welding residual stress adheres to the self-equilibrium criteria of the entire section, but not to the self-equilibrium of individual plates. The plate’s unbalanced force is influenced by section sizes and welding parameters.
2. A larger plate size correlates with a decrease in the residual compressive stress across the section, with a more pronounced reduction observed in adjacent plates. Additionally, augmenting the number of welding passes tends to diminish residual compressive stresses across the section.
3. The membrane residual compressive stress of the unified model advocated by ECCS exhibits an average of 9.4 times the measured values obtained in this study. The models proposed by Ban and Smodi are, on average, 3.5 and 1.5 times, respectively.
4. A 3DTEFEM tailored for Q690 steel welded box sections was developed to conduct parameter analysis, culminating in the development of a precise unified model. It can serve as an accurate residual stress model for the buckling analysis of the compressed member.

However, the computational efficiency of the 3DTEFEM used in this model is relatively low, and the proposed unified residual stress model in this study is specifically designed for Q690 steel welded square sections with a plate thickness of 12 mm. To overcome this limitation, further optimization of the 3DTEFEM is necessary. Additionally, efforts should be made to extend the scope of parameter analysis, allowing for the development of a more widely applicable unified residual stress model.

## Figures and Tables

**Table 1:** Dimensions of the specimens.

Specimen	Statistical Indicator	*H*_b_ (mm)	*B*_b_ (mm)	*t*_x_ (mm)	*t*_s_ (mm)	*b*_b_/*t*_s_	*h*_b_/*t*_x_	*L* (mm)	Number of Welding Passes
RB-1	MV	203.2	204.6	11.94	11.97	15.10	15.01	1142	2
	SD	0.3	0.5	0.03	0.03	0.04	0.03	4	—
RB-2	MV	203.5	324.8	11.95	11.96	25.16	15.03	1621	2
	SD	0.4	0.3	0.02	0.03	0.02	0.03	3	—
RB-3	MV	323.1	204.4	11.91	11.94	15.12	25.12	1620	2
	SD	0.4	0.2	0.05	0.04	0.03	0.03	3	—
RB-4	MV	212.1	264.5	11.93	11.95	20.14	15.78	1378	2
	SD	0.3	0.3	0.02	0.03	0.04	0.01	3	—
RB-5	MV	203.8	204.3	11.93	11.96	15.09	15.08	1142	1
	SD	0.2	0.2	0.04	0.02	0.02	0.04	4	—

**Table 2:** Chemical composition of welding wire (wt.%).

Welding Wire	C	Mn	Si	S	P	Cr	Ni	Mo	Cu	Fe
CHW-80C	0.076	1.86	0.52	0.006	0.007	0.32	2.15	0.60	0.21	Balance

**Table 3:** Mechanical properties of deposited metal.

Tensile Strength (MPa)	Yield Strength (MPa)	Elongation at Failure (%)
820	700	18

**Table 4:** Chemical composition of Q690 steel plates (wt.%).

Steel Plate	C	Mn	Si	S	P	Cr	Ni	Fe
Q690-12	0.08	1.76	0.07	0.001	0.015	0.21	0.02	Balance
Q690-16	0.08	1.74	0.08	0.001	0.01	0.23	0.01	Balance

**Table 5:** Material properties of steel plates.

Steel Plate Number	Statistical Indicator	Yield Strength *f*_y_ (MPa)	Tensile Strength *f*_u_ (MPa)	Young’s Modulus *E* (GPa)	Strain *ε*_st_ (%)	Strain *ε*_u_ (%)	Elongation at Failure (%)	*f*_u_/*f*_y_
Q690-12	MV	834	869	214.90	3.34	6.14	20.08	0.960
	SD	0.68	6.04	7.12	0.18	0.14	0.76	0.006
Q690-16	MV	808	829	205.25	2.83	5.53	19.79	0.975
	SD	2.78	1.28	3.61	0.57	0.20	1.14	0.002

**Table 6:** Unbalanced force.

Specimen	*F*_err_ (kN)					*σ*_err_ (MPa)
	Left Web	Top Flange	Right Web	Bottom Flange	Entire Section	Entire Section
RB-1	116.22	−95.68	92.69	−98.94	14.29	1.55
RB-2	222.96	−174.43	148.15	−202.00	−5.32	−0.44
RB-3	14.23	6.98	13.37	−3.94	30.64	2.53
RB-4	156.68	−161.27	166.77	−146.46	15.73	1.27
RB-5	78.08	−62.77	90.78	−39.38	66.71	7.24

**Table 7:** Simplified membrane residual stress model (unit: MPa).

Specimen	Statistical Indicator	*σ*_src_	*σ*_srt_	*σ*_xrc_	*σ*_xrt_
RB-1	MV	−86.14	278.53	−55.51	402.31
	SD	14.95	48.33	6.29	45.62
RB-2	MV	−75.32	305.44	−46.82	517.18
	SD	11.17	45.32	8.77	96.87
RB-3	MV	−46.7	324.12	−63.75	419.91
	SD	6.49	45.08	14.80	97.48
RB-4	MV	−56.28	102.95	−42.68	413.79
	SD	4.25	7.78	9.98	96.71
RB-5	MV	−108.06	420.83	−81.48	464.54
	SD	12.69	53.47	13.28	66.76

**Table 8:** Comparison of the measured and simulated membrane residual compressive stress.

Specimens	Measured Stress (MPa)		Simulated Stress (MPa)	
	Flange	Web	Flange	Web
RB-1	−86	−56	−83	−59
RB-2	−75	−47	−65	−33
RB−3	−47	−64	−41	−57
RB-4	−56	−43	−59	−21
RB-5	−108	−81	−120	−88

**Table 9:** Unified residual stress model.

Parameter	Value (MPa)
*σ_src_*	$-19.2-39.4\times \exp(-\frac{b_{b}}{11.4\times t_{s}})$
*σ_xrc_*	$-11.9-87.9\times \exp(-\frac{b_{b}}{23.4\times t_{x}})$
*σ_xrt_*	420
*σ_srt_*	Self-equilibrium

Tip: *t*_s_ = *t*_x_ = 12 mm, *b*_b_/*t*_s_ = *h*_b_/*t*_x_.

## Data Availability

Data are contained within the article.

## Abbreviations

HSS: High-strength steel; CMS: Conventional mild steel; FEA: Finite element analysis; 3DTEFEM: Three-dimensional thermal–elastic–plastic finite element model; WEDM: wire-cut electron discharge machine
